# Supplementary material for: CELLoGeNe - An energy landscape framework for logical networks controlling cell decisions
Source: iScience. 2022 Jul 14;25(8):104743. doi: 10.1016/j.isci.2022.104743 (PMC9356104; doi:10.1016/j.isci.2022.104743)
Supplement: Document S1. Table S1 and Figures S1–S6 [file mmc1.pdf]

**iScience, Volume *25***

## Supplemental information

# CELLoGeNe - An energy landscape framework for logical networks controlling cell decisions

**Emil Andersson, Mattias Sjö, Keisuke Kaji, and Victor Olariu**

**Supplementary Table S1, related to STAR ★ METHODS-Configurations of operators.**

Example values of $N(p,k)$ for varying $k$ (columns) and $p$.

| $k$ | 2 | 3 | 4 | 5 | 6 | 10 | 15 | 20 |
|---|---|---|---|---|---|---|---|---|
| $p=2$ | 2 | 8 | 32 | 128 | 512 | 131 072 | $1.3 \times 10^8$ | $1.4 \times 10^{11}$ |
| $p=3$ | 3 | 21 | 147 | 1029 | 7203 | $1.7 \times 10^7$ | $2.9 \times 10^{11}$ | $4.9 \times 10^{15}$ |
| $p=4$ | 4 | 40 | 400 | 4000 | 40 000 | $4.0 \times 10^8$ | $4.0 \times 10^{13}$ | $4.0 \times 10^{18}$ |
| $p=6$ | 6 | 96 | 1536 | 24 576 | 393 216 | $2.6 \times 10^{10}$ | $2.7 \times 10^{16}$ | $2.8 \times 10^{22}$ |

**A**

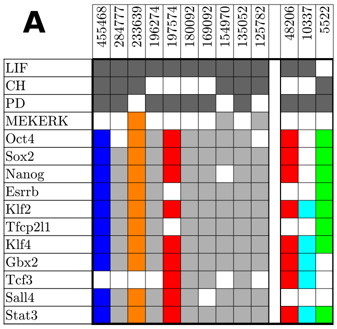


**B**

| Target | Configuration 1 | Configuration 2 | Configuration 3 |
|---|---|---|---|
| LIF | - | - | - |
| CH | - | - | - |
| PD | - | - | - |
| MEKERK | PD | PD | PD |
| Oct4 | (Sox2 ↑ Klf2 ) ⊼ Esrrb) | (Sox2 ↓ Klf2 ) ⊼ Esrrb) | (Sox2 ⊽ Esrrb) ⩡ Klf2 |
| Sox2 | Nanog ↑ Sall4 | Nanog ⊽ Sall4 | Nanog ↑ Sall4 |
| Nanog | (MEKERK ↑ Klf2) ⊼ Oct4 | (MEKERK ⩟ Klf2) ⊽ Oct4 | MEKERK ↑ (Klf2 ⊽ Oct4) |
| Esrrb | (Nanog ⩟ Tcf3) ⩡ Tfcp2l1 | (Nanog ⊼ Tfcp2l1) ⊽ Tcf3 | (Nanog ⩡ Tfcp2l1) ⩡ Tcf3 |
| Klf2 | Sall4 ⩡ Klf4 | Sall4 ↑ Klf4 | Sall4 ⩡ Klf4 |
| Tfcp2l1 | (Klf4 ⩟ (Esrrb ⩡ Oct4)) ⊽ (Stat3 ⩡ Tcf3) | (Stat3 ⊼ Esrrb) ⊽ ((Oct4 ⩟ Klf4) ⊽ Tcf3) | ((Stat3 ⊽ (Esrrb ⩟ Oct4)) ⩟ Klf4) ⩡ Tcf3 |
| Gbx2 | Stat3 | Stat3 | Stat3 |
| Klf4 | Stat3 ↑ Gbx2 | Stat3 ↑ Gbx2 | Stat3 ↑ Gbx2 |
| Tcf3 | CH ⊼ MEKERK | CH ⩟ MEKERK | CH ↑ MEKERK |
| Sall4 | Tfcp2l1 | Tfcp2l1 | Tfcp2l1 |
| Stat3 | LIF | LIF | LIF |

## Supplementary Figure S1, related to Figure 3.

## CELLoGeNe results from random search of the configuration space for the GRN controlling maintenance of pluripotency.

(**A**) Number of times the 10 most common attractors and experimental constraints occur in the $10^6$ calculated energy landscapes. (**B**) The 3 configurations with the lowest degeneracy out of the 163 valid configurations found when trying $10^6$ configurations.

**A**

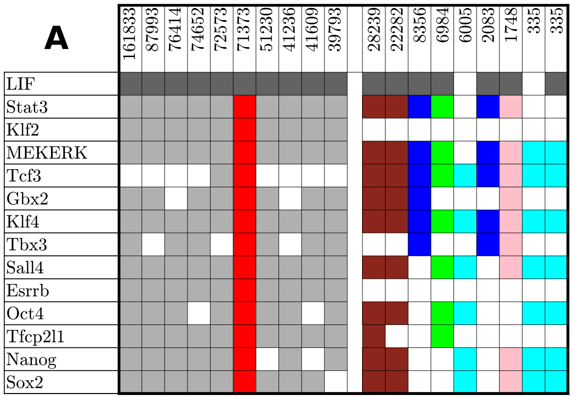


**B**

| Target | Configuration 1 | Configuration 2 |
|---|---|---|
| LIF | - | - |
| Stat3 | LIF | LIF |
| Klf2 | (Esrrb ⊼ Klf4) ⊼ Tcf3 | Klf4 ↓ (Tcf3 ⊽ Esrrb) |
| MEKERK | LIF | LIF |
| Tcf3 | MEKERK ⊼ Klf4 | MEKERK ↑ Klf4 |
| Gbx2 | Sox2 ⊼ (Stat3 ⊼ Oct4) | Stat3 ↓ (Oct4 ↑ Sox2) |
| Klf4 | (Gbx2 ⊻ Stat3) ⊽ Klf2 | Stat3 ↓ (Gbx2 ⊼ Klf2) |
| Tbx3 | Tcf3 ↑ ((Klf4 ↓ Oct4) ↑ Esrrb) | Oct4 ⊽ (Tcf3 ⊼ (Klf4 ↓ Esrrb)) |
| Sall4 | Sox2 ⊽ Tfcp2l1 | Sox2 ↑ Tfcp2l1 |
| Esrrb | Tcf3 ↓ (Nanog ↓ (Sall4 ↑ Tfcp2l1)) | (Tfcp2l1 ⊼ Sall4) △ (Tcf3 ⊽ Nanog) |
| Oct4 | Sall4 ↓ Tcf3 | Tcf3 ⊼ Sall4 |
| Tfcp2l1 | Stat3 ↓ (Esrrb ↑ (Oct4 ↑ Tcf3)) | (Oct4 ⊻ (Stat3 ↓ Esrrb)) ⊽ Tcf3 |
| Nanog | MEKERK ↑ ((Oct4 ⊻ Sall4) ⊼ Sox2) | (MEKERK ↓ Oct4) ⊽ (Sox2 ↓ Sall4) |
| Sox2 | Nanog ⊽ (MEKERK ⊻ (Sall4 ↓ (Tcf3 ⊼ Tbx3))) | ((Tcf3 △ (Sall4 ⊼ MEKERK)) ↓ Nanog) △ Tbx3 |

**Supplementary Figure S2, related to Figure 4.**

**CELLoGeNe results from random search of the configuration space for the GRN controlling reprogramming from MEF to iPSC.**

(**A**) Number of times the 10 most common attractors and the attractors in Figure 4C occur in the $10^6$ calculated energy landscapes. (**B**) The 2 valid configurations found when trying $10^6$ configurations.

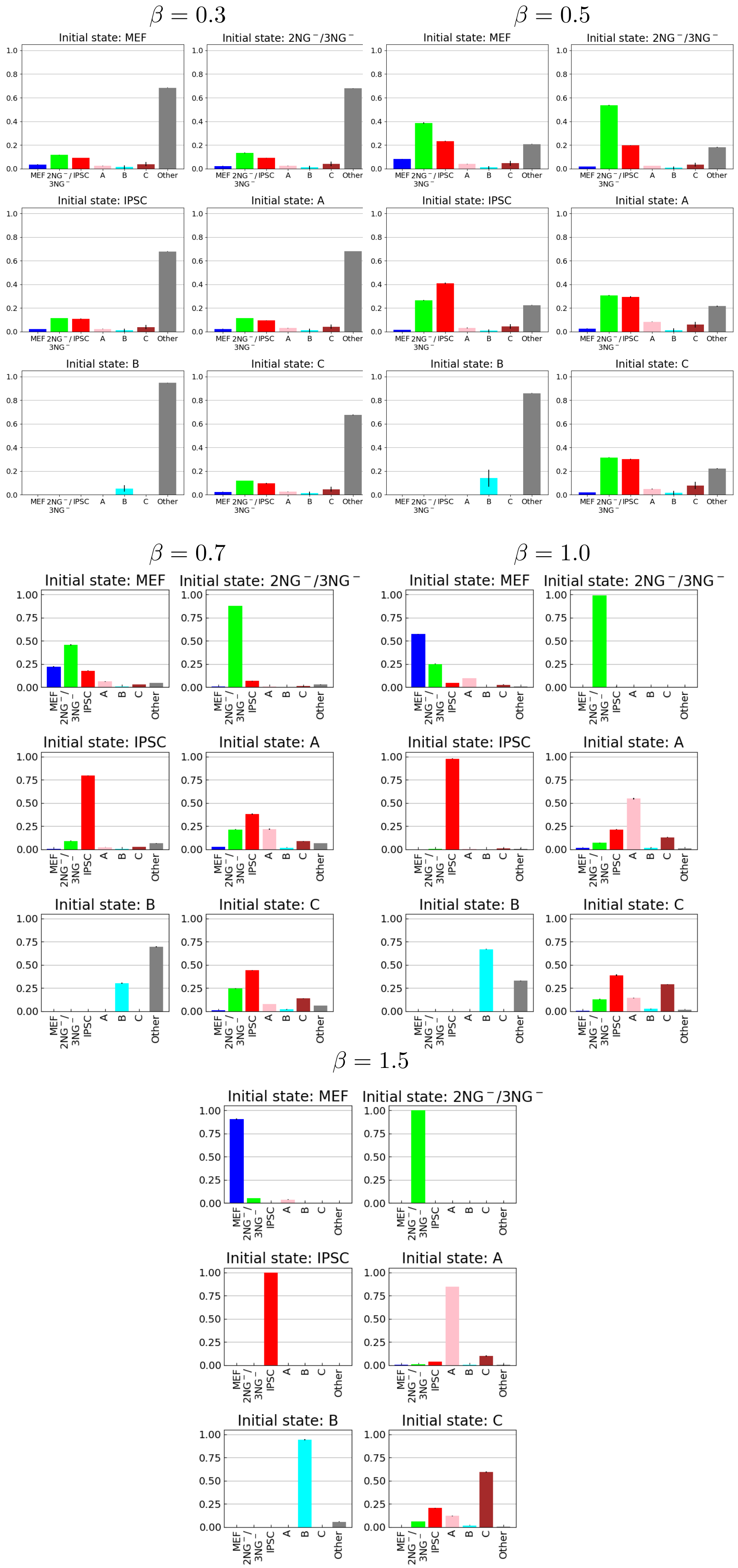


**Supplementary Figure S3, related to Figure 4.**
**Simulated reprogramming with noise**

Simulated reprogramming with different values on the noise level $\beta$. 10 000 cells were initialised in each initial state and the simulations were repeated three times. Error bars indicate the standard deviation.

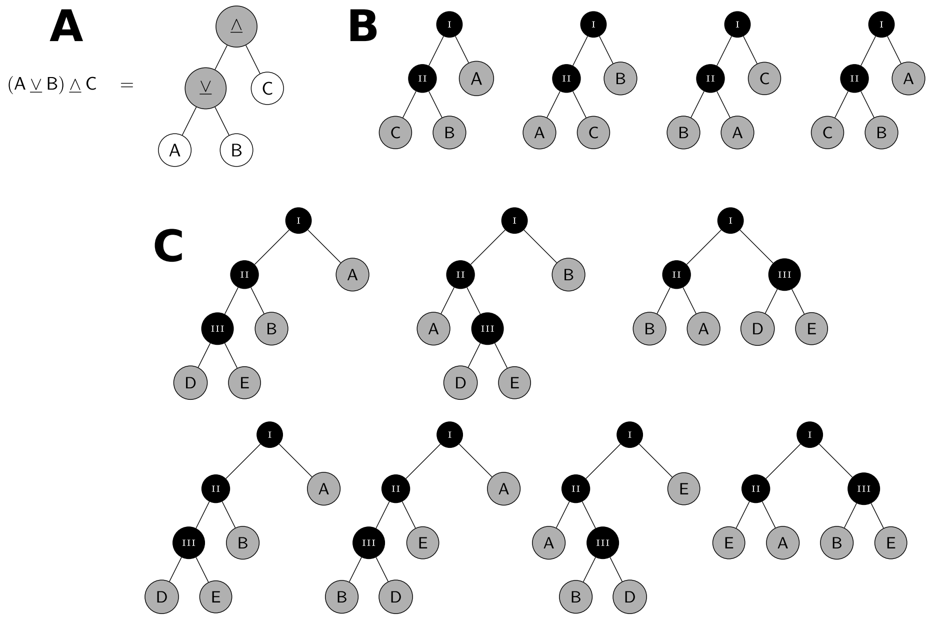


**Supplementary Figure S4, related to STAR ★ METHODS-Testing configurations of operators.**

**Tree Representation and Permutation Demonstration of Gene Input Configurations**

(**A**) A configuration of three genes represented as a binary tree. (**B**) Demonstration of the permutation of a tree. The black nodes are operator nodes, while the grey nodes can be either leaves or operator nodes with subtrees attached. (**C**) An extension of **B**, where node C is replaced with III and is revealed to have two children, D and E. In the top row, **B** is simply repeated. After that, a single permutation is performed around Node II, followed by the beginning of the next permutation cycle of node I, demonstrating how node E travels up the tree (node 4 will as well, later) and how the entire subtree with B and D is moved.

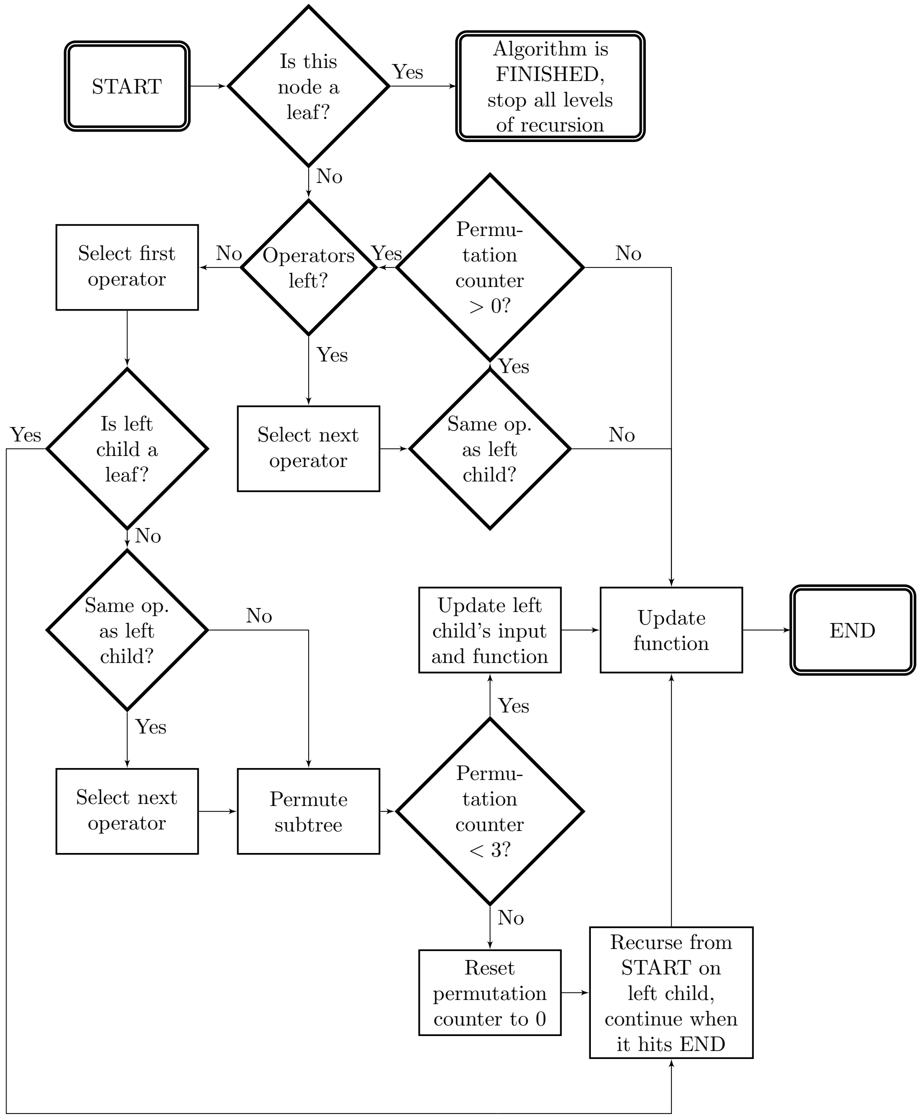


**Supplementary Figure S5, related to STAR ★ METHODS-Testing configurations of operators.**

**Operator Configuration Stepping Algorithm**

Flowchart describing the algorithm for trying all configurations of a gene. Applying it on the root once moves the tree to the next configuration. To save space, "op." is used as an abbreviation of "operator".

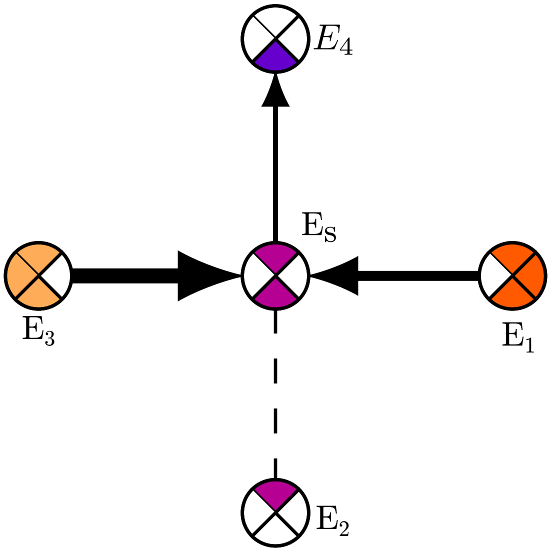


**Supplementary Figure S6, related to STAR ★ METHODS-Marble simulations.**
**Energy Dependent Transition Update in Marble Simulations**
Example state $s$ has energy $E_s$ and its four neighbours have energies $E_{1...4}$. Since state 4 is the only neighbour with energy $E_4 < E_s$ the transition $s \to 4$ is the most probable, although all the other transitions are also possible due to the stochasticity. The probability is determined by the noise level $\beta$ and energy differences between the states.
